# Reconstituted HDL ameliorated renal injury of diabetic kidney disease in mice

**DOI:** 10.14814/phy2.16179

**Published:** 2024-08-06

**Authors:** Yu Tao, Andras G. Lacko, Nirupama A. Sabnis, Paromita Das‐Earl, Deena Ibrahim, Nicole Crowe, Zhengyang Zhou, Mark Cunningham, Angie Castillo, Rong Ma

**Affiliations:** ^1^ Department of Physiology and Anatomy University of North Texas Health Science Center Fort Worth Texas USA; ^2^ Department of Population and Community Health University of North Texas Health Science Center Fort Worth Texas USA

**Keywords:** albuminuria, diabetic kidney disease, eNOS^−/−^ *dbdb* mouse, glomerular filtration rate, rHDL

## Abstract

Diabetic kidney disease (DKD) is a devastating kidney disease and lacks effective therapeutic interventions. The present study was aimed to determine whether reconstituted high‐density lipoprotein (rHDL) ameliorated renal injury in eNOS^−/−^ *dbdb* mice, a mouse model of DKD. Three groups of mice, wild type C57BLKS/J (non‐diabetes), eNOS^−/−^ *dbdb* (diabetes), and eNOS^−/−^ *dbdb* treated with rHDL (diabetes+rHDL) with both males and females were used. The rHDL nanoparticles were administered to eNOS^−/−^ *dbdb* mice at Week 16 at 5 μg/g body weight in ~100 μL of saline solution twice per week for 4 weeks via retroorbital injection. We found that rHDL treatment significantly blunted progression of albuminuria and GFR decline observed in DKD mice. Histological examinations showed that the rHDLs significantly alleviated glomerular injury and renal fibrosis, and inhibited podocyte loss. Western blots and immunohistochemical examinations showed that increased protein abundances of fibronectin and collagen IV in the renal cortex of eNOS^−/−^ *dbdb* mice were significantly reduced by the rHDLs. Taken together, the present study suggests a renoprotective effect of rHDLs on DKD.

## 1 INTRODUCTION

Diabetic kidney disease (DKD) is one of the most common complications of diabetes mellitus and is the leading cause of end stage renal disease (Kanwar et al., 2005, 2008; Kasinath, 2012). Early features of DKD include renal/glomerular hypertrophy, glomerular hyperfiltration (elevated glomerular filtration rate, GFR), podocyte injury, and expansion of the glomerular mesangium and deposition of the extracellular matrix proteins. If not controlled, the affected kidneys eventually develop glomerulosclerosis, tubular atrophy, renal fibrosis and renal insufficiency indicated by a declined GFR (Mason & Wahab, 2003; Simonson, 2007; Ziyadeh & Sharma, 1995). However, there are no known therapies currently available that can treat the progressive lesion of renal histology and loss of renal function in DKD. Despite the increased use of antihypertensive medications and renin‐angiotensin system inhibitors in the patients with DKD, the improvement of renal function is modest (Pavkov et al., 2009). New and effective treatments would, therefore, be a significant advance.

High density lipoprotein (HDL) has been described as having potent anti‐inflammatory and antioxidative properties (Gao et al., 2022; Kronenberg, 2018; Murphy et al., 2012; Wolkowicz et al., 2021). Inflammation and oxidative stress are known to contribute to the onset and development of diabetes mellitus and its complications, including DKD (Kanwar et al., 2005, 2008). However, in the context of diabetes, the functional quality of HDL is reduced (Wolkowicz et al., 2021) and thus the protective effects from HDL are compromised. Therefore, raising levels of functional HDL is an important target for reducing the development of diabetes and diabetic complications. Although HDL has been found to be beneficial to the injured kidneys (Moreira et al., 2014), some clinical, epidemiologic, and genetic observations did not show an association of HDL levels with cardiovascular risk and chronic kidney diseases (Kronenberg, 2018). The discrepancies are probably derived from a very large number of proteins and lipids contained in HDL particles, which may play different and even opposite roles (Kronenberg, 2018). Therefore, during recent years, there has been a major shift of focus toward the functionality of HDL particles and the role of its components. In this regard, apolipoprotein A‐I (apoA‐I) which is the major apolipoprotein of almost all plasma HDLs and possesses the beneficial effects of HDL, has been a major target (Gao et al., 2022; McDonald et al., 2003; Sabnis et al., 2012; Wolkowicz et al., 2021). While HDL and apoA‐I supplementation may correct the diabetic complications due to low levels of HDL and protect kidney from injury, isolation of large quantities of HDL and apoA‐I from human plasma that are functionally active is not practical. Therefore, there is a need to design reconstituted/synthetic HDL (rHDL) and apoA‐I mimetics. Over years, rHDLs which mimic the structure and/or function of native HDL, have attracted increasing attention in the treatment and drug delivery for a wide range of disorders (Gao et al., 2022; Sabnis et al., 2012; Shahzad et al., 2011; Wolkowicz et al., 2021). Several infusible rHDLs, including the ones from our group have already been tested in humans (Karalis & Jukema, 2018; Ossoli et al., 2021; Sabnis et al., 2012; Shahzad et al., 2011). However, whether rHDLs can protect kidney from injury in the settings of DKD has never been investigated. The aim of the present study was to determine whether the rHDL treatment could improve renal histology and function of eNOS^−/−^ *dbdb* mice, a well‐established mouse model of DKD (Brosius et al., 2009; Ma et al., 2019; Zhao et al., 2006).

## 2 MATERIALS AND METHODS

### 2.1 Animal preparation

In this study, we used eNOS^−/−^ *dbdb* mice on genetic background of C57BLKS/J as the model of DKD, and their wild type (WT) C57BLKS/J mice as non‐diabetes controls. The mouse model of double knockout of eNOS and leptin receptor (eNOS^−/−^ *db/db*) is a well‐accepted type II diabetes model (Brosius et al., 2009). The eNOS^−/−^ *db/db* mice have many advantages for studying DKD over other models of diabetes because they develop significant albuminuria, decreased GFR, mesangial expansion, glomerular basement membrane thickening, arteriolar hyalinosis, glomerulosclerosis, and tubulointerstitial injury, all of which are features of DKD in humans (Brosius et al., 2009; Ma et al., 2019; Zhao et al., 2006).

A total of 7 WT C57BLKS/J mice (all male) and 18 eNOS^−/−^ *dbdb* mice (9 male and 9 female) were used in the present study. The number of mice used for different analysis presented in this study was based on experimental design and quality of samples collected. All C57BLKS/J mice were purchased from the Jackson Laboratory (Bar Harbor, ME. Stock #: 000662). eNOS^−/−^ *db/db* mice were generated from homozygous eNOS^−/−^ and heterozygous Lepr^db^ (eNOS^−/−^ *db*) on C57BLKS/J background. The eNOS^−/−^ *db* breeders were purchased from The Jackson Laboratory (JAX# 8340, Bar Harbor, ME) and the genotypes of pups were identified by genotyping at age of 3 weeks using High Resolution Melting PCR protocol provided by the vendor and publications in (Mohan et al., 2008; Shesely et al., 1996). The age of mice for experiments ranged from 16 to 20 weeks. All mice were euthanized by cervical dislocation under anesthesia with intraperitoneal injection of ketamine (80–100 mg/kg body weight, Covertus, Catalog#: B8U4‐20036581) + xylazine (5–10 mg/kg body weight, Covertus, Catalog#: NDC: 11695‐4024‐1). All procedures were approved by the University of North Texas Health Science Center (UNTHSC) Institutional Animal Care and Use Committee. All the mice were maintained at the UNTHSC animal facility under local and NIH guidelines. These mice were housed in a specific pathogen‐free facility with a temperature‐controlled room, and regulated with 12‐h light/dark cycle and free access to water and food (standard chow diet: LabDiet, St. Louis, MO) containing 25.1% fiber, 0.29% Na^+^, 19.3% protein, 13.5% fat, and 15.8% calories from fat.

### 2.2 rHDL formulation and delivery

rHDL nanoparticles were formulated as described in (Sabnis et al., 2017) with some modifications. In brief, phosphatidylcholine (Millipore sigma, catalog # P3556) at 7.5 mg/mL, free cholesterol (Millipore sigma, catalog # C8667) at 0.175 mg/mL and cholesteryl oleate (Millipore sigma, catalog # C9253) at 0.075 mg/mL were added to a glass vial, and mixed thoroughly. This mixture was then dried under nitrogen. Apo‐A1 (custom made, MC lab, San Francisco, CA) at 2.5 mg/mL was added to the mixture dropwise and mixed by pipetting. After adding 7.5 mg/mL sodium cholate, phosphate buffer saline (PBS) was used to make up the desired volume. The mixture was mixed thoroughly, incubated at 4°C overnight. It was then dialyzed against 2 L of 1× phosphate‐buffered saline for 48 h, with a change of buffer three times every 2 h on the 1st day, and later kept overnight. The mixture was centrifuged at 1500 rpm for 1 min and then filtered through a 0.22‐μM syringe filter. The solution was characterized for its size, zeta potential and polydispersity index (PDI) to ensure the quality of the nanoparticles using ultra particle analyzer, Malvern Panalytical, UK. The formulations were diluted with filtered PBS pH 7.4, for these measurements. The machine captured an average of 100 runs and the number distribution of average particle size was reported. The zeta potential measurements were performed by dispersing the particles in an aqueous solution at 25°C with a scattering angle of 90° in Malvern 1070 folded capillary cartridges. The chemical composition of the rHDL nanoparticles was performed using cholesterol and phospholipids were determined by respective enzymatic reagent kits (cholesterol E and phospholipid C), obtained from Wako Pure Chemical Industries Ltd, Richmond, VA. Using microtiter plate assays as per manufacturer's suggestions. Protein determinations were carried out using a bicinchoninic acid (BCA) protein assay kit, from Thermo Scientific, Rockford, IL (Thermo Scientific™ Pierce™ BCA protein assay kit, catalog # PI23227). The nanoparticles were stored at −20°C until used. All reagents used for the rHDL preparation were purchased from sigma‐Aldrich (St Louis, MO, USA). All organic chemicals and solvents used were of reagent grade. Apo‐A1 was obtained from MC lab (San Francisco, CA, USA).

The formulated rHDL nanoparticles were given to eNOS^−/−^ *dbdb* mice at age of 16 weeks at 5 μg/g body weight (BW) in ~100 μL of phosphate‐buffered saline solution via retro‐orbital injection every 3 days (two times a week) for 4 weeks. This dose and treatment regimen were based on the recent publication using CER‐001 (containing the similar ingredients to our rHDL) in mice (Ossoli et al., 2021) and our pilot study. Before and after the 4‐week treatment, GFR was assessed and fasting blood samples and urinary samples were collected for blood glucose and urinary albumin excretion assay. Mice were sacrificed after completion of the rHDL treatment for renal cortical protein extraction and renal histology/pathology examinations.

### 2.3 Transdermal measurement of GFR

GFR was measured in conscious, freely moving mice using transdermal measurement of FITC‐sinistrin clearance rate as previously described (Chaudhari et al., 2022; Tao, Young‐Stubbs, et al., 2023). Anesthetized mice were positioned on a surgery plate and the right dorsal hair was shaved with an electrical shaver followed by the application of depilatory lotion and 70% ethanol to remove any residual hair. The transdermal fluorescence detector (MB 0309 Mini, MediBeacon Inc., Germany) was directly attached to the naked skin and fixed to the mouse body using medical tape. FITC‐conjugated sinistrin (Fresenius‐Kabi Austria, Austria. Catalog#: VE17193321) was then administered by retro‐orbital injection at 0.03 mg/g BW (30–50 μL) using a 0.5 mL BD insulin syringe (18G × ½). The excitation kinetics of the exogenous GFR tracer were recorded using the software provided by the vendor (MB Lab Ver. 2.18) in freely‐moving, conscious mice for 1.5 h. The recorded sinistrin clearance curve was fitted by the software provided by the vendor (MB Studio Ver. 2.1) using a two‐compartment model. GFR was calculated based on the half‐life (*t*_1/2_) of plasma FITC‐sinistrin decay using the formula 14616.8/(*t*_1/2_) and reported as μL/min/100 g BW as previously described (Scarfe et al., 2018; Schock‐Kusch et al., 2013; Schreiber et al., 2012).

### 2.4 Measurement of blood glucose (BG)

Fasting BG was evaluated with a LifeScan One Touch glucometer (Johnson & Johnson, Milpitas, CA) by tailed blood sampling from conscious mice at 2:00 p.m. after fasting for 4 h initiated at 10:00 a.m.

### 2.5 Measurement of blood urea nitrogen (BUN)

Blood samples were collected from retro‐orbital veins under anesthesia with intraperitoneal injection of ketamine plus xylazine (100 mg/kg + 10 mg/kg). Serum samples were stored at −80°C freezer until used. BUN was measured using QuantiChrom™ Urea Assay kit (DIUR‐100, BioAssay System, Hayward, CA. Catalog#: DIUR‐100) following the protocol provided by the manufacturer.

### 2.6 Measurements of urine output (UO) and urinary albumin excretion

Twenty‐four hour urine samples were collected through metabolic cages (Catalog #: 370 0M022, Braintree Scientific Inc., Braintree, MA). Urinary albumin and creatinine excretion were determined using Albuwell M kits (Exocell, Philadelphia, PA. Catalog#: NC9182134 for Albumin and NC9129340 for Creatinine). Albumin excretion rate was expressed as the ratio of urinary albumin concentration to urinary creatinine concentration (ACR, μg/mg).

### 2.7 Measurements of antioxidant capacity

Urine antioxidant capacity was measured using the Antioxidant Assay Kit (Catalog #: 709001, Cayman Chemical, Ann Harbor, MI), according to the manufacturer's instruction. Briefly, urine samples were collected from mice to assess total antioxidant capacity via the inhibition of the conversion of 2,2′‐azino‐bis(3‐ethylbenz‐thiazoline‐6‐sulfonic acid) (ABTS) in the presence of hydrogen peroxide (H_2_O_2_) to oxidized ABTS (ABTS+) by presence of endogenous antioxidants. Trolox standards, a water soluble analog of the Vitamin D antioxidant, were used to calculate the endogenous antioxidant capacity. Urine samples were diluted to a 1:10 working concentration and duplicates were added to each well for measurements on the 96‐well plate via spectrophotometry. Urine antioxidant capacity was compared to Trolox standards of 0.068, 0.135, 0.203, 0.270, 0.338, and 0.495 mM Trolox. The antioxidant capacity was quantified as millimolar Trolox equivalents (mM Trolox) at 405‐nm wavelength.

### 2.8 Renal tissue preparation

Mice were anesthetized by intraperitoneal injection of ketamine with xylazine (100 + 10 mg/kg). After perfusion with physiological saline solution through the left ventricle to wash out blood, the left kidneys were removed and were immediately snap‐frozen for extracting renal cortical proteins. The mice were then perfused with 4% paraformaldehyde and the right kidneys were excised and decapsulated. The right kidneys were cut in half through a midsagittal plane and were fixed with 4% paraformaldehyde. The fixed kidneys were dehydrated through a graded series of ethanol, infiltrated and embedded in paraffin, sectioned (~3 μm), and mounted on a glass slide for histological and immunohistochemistry examinations.

### 2.9 Isolating renal cortex and extracting cortical proteins

The renal cortex was separated from the other regions of the kidney and then minced by sharp blades. The minced cortical tissue was then sonicated in a lysis buffer followed by centrifugation at 21000*g* for 15 min at 4°C. The supernatants were collected for Western blot.

### 2.10 Western blot

Proteins were separated by 7.5% Mini‐Protean TGX stain‐free precast gels (Bio‐Rad, Cat#: 456‐8024), transferred to PVDF membranes, and probed with primary antibodies against fibronectin (FN) (rabbit polyclonal, catalog#: ab2413, lot#: 1007002‐1, abcam, 1:500 dilution), collagen IV (Col IV) (rabbit polyclonal, catalog#: ab6586, lot#: 1034101‐2, abcam, at 1:500 dilution) and GAPDH (EMD Millipore, Cat#: MAB374, lot#: 3140165, at 1:300 dilution). Bound antibodies were visualized with Super Signal West Femto (Catalog#: 34095) or Pico Luminol/Enhancer Solution (Catalog#: 3458) (Thermo Scientific, Rockford, IL). The specific protein bands were visualized and captured using the AlphaEase FC Imaging System (Alpha Innotech, San Leandro, CA). The integrated density value (IDV) of each band was measured by drawing a rectangle outlining the band using AlphaEase FC software with auto background subtraction. Abundance of the target proteins was evaluated by normalizing the IDV of their bands to that of the GAPDH band on the same blot. All of the primary antibodies were validated by manufacturers.

### 2.11 Histological analysis and assessment of glomerular injury

Periodic acid‐Schiff (PAS) staining was performed to evaluate glomerular histology and glomerulosclerosis. Three‐micrometer paraffin–embedded kidney sections were stained with PAS (Sigma‐Aldrich, St. Louis, MO. Catalog#: 395B). Images were captured using an Olympus DP70 digital camera with DP manager software (version 2.2.1). The glomerular injury scores in PAS‐stained sections were graded by a blind observer, using a scale of 0–4: 0 was assigned to normal glomeruli; 1 was assigned to glomeruli with mesangial expansion; 2 was assigned to glomeruli in which sclerosis encompassed less than 50% of the glomerulus; 3 was assigned to glomeruli with lesions encompassing 50%–75% of the glomerulus; and 4 was assigned to glomeruli with lesions encompassing more than 75% of the glomerulus, or fully collapsed glomeruli. For each mouse, glomerular score was evaluated in over 50 glomeruli from 5 sections (>10 glomeruli/section) and individual scores were averaged to obtain a mean value for that mouse.

### 2.12 Masson's trichrome staining

Kidney paraffin sections (~3 μm thickness) were stained with Masson‐trichrome to evaluate the severity of renal fibrosis. The staining reagents were purchased from Sigma (St. Louis, MO. Catalog#: HT10312) and staining was performed according to the protocol provided by the manufacturer. Images were captured using an Olympus DP70 digital camera with DP manager software (version 2.2.1). For determining areas of renal fibrosis, RGB color‐images were opened in ImageJ software and user‐defined vectors were input in the color deconvolution feature of ImageJ plugin to adequately separate red, blue and green components of the 3 different dye colors from the stained images. A low and high threshold value for the blue color (fibrotic area) in the images was stringently defined and a binary 8‐bit image was generated. Fibrotic areas were quantified as % area with blue staining by an observer blinded to the groups.

### 2.13 Immunohistochemical staining

After deparaffinization of kidney sections, antigen retrieval was achieved by heating the sections in 10 mM citrate buffer in a microwave for 10 min. The sections were blocked by 5% goat serum (Catalog No. 005000121, Jackson Immuno Research Labs) for 30 min at room temperature and then incubated with anti‐Col IV antibody (rabbit polyclonal, catalog #: ab6586, lot #: 1034101–2, Abcam) at 1:100, or anti‐FN antibody (rabbit polyclonal, catalog #: ab2413, lot #: 1007002‐1, Abcam) at 1:100, or anti‐WT1 polyclonal rabbit antibody (MyBioSource Cat. No. MBS9203569) at 1:20 at 4°C overnight. The sections were incubated with anti‐rabbit poly HRP IHC reagent (Catalog #: IHC‐2291, General Bioscience Corporation) at room temperature for 1 h, followed by incubation with peroxidase substrate solution (DAB substrate kit, Peroxidase, Vector Laboratories, Cat#: SK‐4100; Lot#: ZG0526) for 2–3 min, stained in hematoxylin solution (Statlab, SKU#: SL401; Lot#: 161917) for 15 seconds, dehydrated with ethanol at 80%, 90%, and 100% (5 min each) followed by Histoclear II for 5 min. The slide was then placed a coverslip with DPX mounting medium (Cat#: 13510; Lot#: 200626‐16). Sections were examined using an Olympus microscope (BX41) and an Olympus DP70 digital camera with DP manager software (version 2.2.1). Images were uniformly adjusted for brightness and contrast and positive staining area was identified by ImageJ color deconvolution plugin and analyzed using ImageJ (version 1.50b; NIH) (normalized to the whole area of the glomerulus), which was conducted by a blinder observer.

### 2.14 Statistical analysis

Data were reported as means ± SD. The one‐way ANOVA plus Student–Newman–Keuls post‐hoc analysis or Kruskal‐Wallis One Way ANOVA on Ranks plus Dunn's post‐hoc analysis or One Way Repeated Measures ANOVA followed by Bonferroni t‐test analysis or Student's unpaired *t*‐test was used to analyze the differences among groups or between two groups. Two way repeated measures ANOVA followed by Bonferroni *t*‐test was conducted for analyzing the differences before and after treatment among multiple groups (Figure 2c). All statistical analyses were stated in each figure legend. Two‐sided *p* < 0.05 was considered statistically significant. Statistical analyses were performed using SigmaStat (Jandel Scientific, San Rafael, CA).

## 3 RESULTS

### 3.1 BW, BG, UO, and kidney weight (KW) in WT, eNOS ^−/−^ *dbdb*, and eNOS ^−/−^ *dbdb* treated with rHDL mice

Three groups of mice were included in this study, that is, WT C57BLKS/J (non‐diabetic, non‐Db), eNOS^−/−^ *dbdb* (diabetic, Db), and eNOS^−/−^ *dbdb* mice treated with rHDL (Db+rHDL). rHDL treatment was applied to Db mice at 5 μg/g BW in ~100 μL of phosphate buffer saline solution via retro‐orbital injection twice per week at Week 16 for 4 weeks.

We have previously reported that there were no sex differences in BG level, UO and renal histological and functional injury except BW in either WT C57BLKS/J or eNOS^−/−^ *dbdb* mice (Ma et al., 2019). Therefore, in this study we pooled male and female mice in the same group for all experiments except for BW data analysis, of which we only included male mice.

**FIGURE 1:**
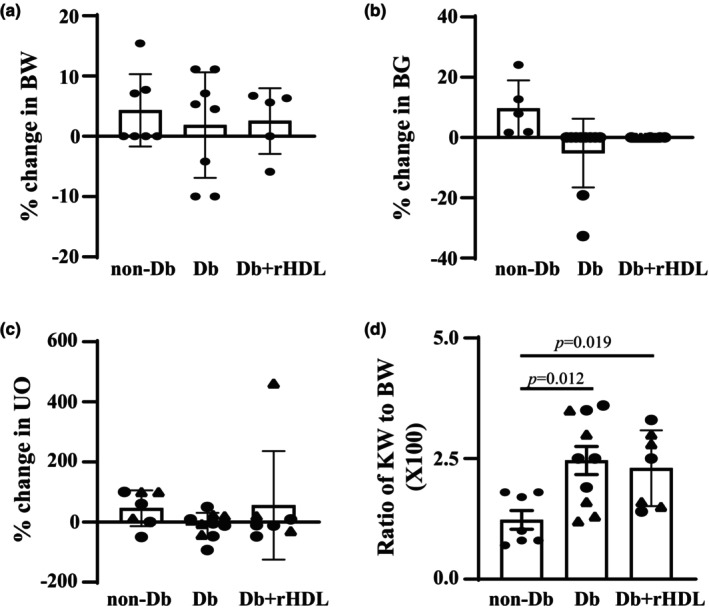
Influence of rHDL treatment on BW (a), BG level (b), UO (c), and ratio of KW to BW (d) in mice with DKD. (a–c) show the percent changes in BW, fasting BG, and 24‐h UO from the age of 16 weeks to 20 weeks, calculated by (the values at Week 16/the values at Week 20) ×100%. (d) shows the ratio of KW to BW at Week 20 when mice were sacrificed. In Db+rHDL group, eNOS^−/−^ *dbdb* mice (Db mice) were treated with rHDL at 5 μg/g BW in 100 μL of phosphate buffer saline solution via retro‐orbital injection twice per week from Week 16 for 4 weeks. Statistics: One way ANOVA followed by Student–Newman–Keuls post‐hoc analysis. In (a), 7 non‐Db, 8 Db, and 5 Db+rHDL mice (all males) were included. In (b), 5 non‐Db (all males), 10 Db (males), and 8 Db+rHDL (4 males and 4 females) mice were used. In (c), 7 non‐Db (4 males and 3 females), 10 Db (6 males and 4 females), and 7 Db+rHDL (4 males and 3 females) mice were included. In (d), 7 non‐Db (all males), 10 Db (5 males and 5 females), and 8 Db+rHDL (4 males and 4 females) mice were included. Solid circle represents males and solid triangle represents females.

Table 1 listed BW, fasting BG levels and 24‐h UO in non‐Db, Db, and Db+rHDL mice measured at ages of 16 weeks (16 W, before treatment) and 20 weeks (20 W, after treatment). In agreement with our previous report (Ma et al., 2019), Db mice were significantly heavier than non‐Db mice (Table 1). There was no significant difference in baseline BW between Db and Db+rHDL mice (Table 1). Although BW in non‐Db and Db mice increased 4 weeks later (at Week 20), the percentages of increase between the two groups were not significantly different (Figure 1a). rHDL treatment (Db+rHDL) did not have a significant influence on the growth of Db mice (Figure 1a).

**TABLE 1:** Physiological parameters of different groups of mice at 16 weeks (16 W) and 20 weeks (20 W).

Group	BW (g)		BG (mg/dL)		UO (μL/h)	
	16 W	20 W	16 W	20 W	16 W	20 W
non‐Db	28.6 + 2.8	29.7 + 2.1 (*n* = 7)	130.6 + 13.7	144 + 26.2 (*n* = 5)	13.7 + 9.5	15.8 + 10.2 (*n* = 7)
Db	38.8 + 5.9***	39.3 + 5.3 (*n* = 8)	640.7 + 29.4*	610 + 91.4 (*n* = 10)	1051.8 + 407.9**	860.6 + 450.4 (*n* = 10)
Db+rHDL	34.0 + 3.2*	34.8 + 3.0 (*n* = 5)	650.0 + 0.0*	650 + 0.0 (*n* = 8)	994.6 + 469.5**	942.6 + 416.3 (*n* = 7)

*Note*: Data are expressed as mean ± SD. Compared to non‐Db group at 16 W. <*n* = Inside parenthesis indicates the number of mice in each group.

* Denotes *p* < 0.05.

** Denotes *p* < 0.01.

*** Denotes *p* < 0.001.

Similarly, fasting BG levels and 24‐h UO were significantly elevated in eNOS^−/−^ *db/db* mice compared to C57BLKS/J mice at Week 16 (Table 1). There were no significant changes in BG level and urine volume in non‐Db and Db mice throughout the study period (Figure 1b,c). The baseline fasting BG and UO of Db+rHDL mice (before rHDL treatment at Week 16) were comparable to those of Db mice (Table 1). rHDL treatment for 4 weeks did not significantly change the hyperglycemia and diuresis of Db mice (Figure 1b,c).

Renal hypertrophy is one feature of early DKD. eNOS^−/−^ *db/db* mice showed marked renal hypertrophy, indicated by a significant increase in the ratio of KW to BW. rHDL treatment for 4 weeks did not improve renal hypertrophy in Db mice (Figure 1d).

### 3.2 rHDL treatment ameliorated albuminuria and blunted GFR decline without improving elevated BUN in mice with DKD

**FIGURE 2:**
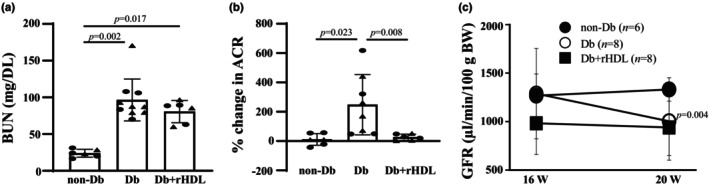
BUN (a), albumin excretion rate (b), and GFR (c) in WT C57BLKS/J (non‐Db), eNOS^−/−^ *dbdb* (Db) and eNOS^−/−^ *dbdb +* rHDL treatment (Db+rHDL) mice. (a) BUN levels at Week 20. (b) Percent changes in albumin excretion rate indicated by ACR from Week 16 to 20. The percentages were calculated by [(the ACR at Week 20–ACR at Week 20)/ACR at Week 16] × 100%. (c) Calculated GFR for individual mice in non‐Db, Db and Db+rHDL mice at Week 16 and Week 20. In (a, b), statistical analysis used One Way ANOVA on ranks followed by Dunn's post‐hoc analysis. Solid circle represents males and solid triangle represents females. In (c), statistical analysis used two way repeated measures ANOVA followed by Bonferroni *t*‐test analysis. The non‐Db group included 6 mice (4 males and 2 females) for all measurements. Db group included 10 mice (4 males and 6 females) in (a) and 8 mice (4 males and 4 females) in (b, c). Db+rHDL group included 6 mice (3 males and 3 females) in (a, b), but 8 mice (4 males and 4 females) in (c).

To examine whether rHDL had renoprotection on DKD, we assessed BUN level, albumin excretion rate, and GFR in non‐Db and Db mice with and without rHDL treatment. The levels of BUN at Week 20 were significantly elevated in Db mice compared to non‐Db mice (Figure 2a). Treatment of Db mice with rHDL for 4 weeks starting from Week 16 did not significantly improve the elevated BUN (Figure 2a). In agreement with our previous report (Ma et al., 2019), albumin excretion rate was significantly increased in eNOS^−/−^ *dbdb* mice compared to non‐Db controls at Week 16 (Table 2). After 4 weeks (at Week 20), the albuminuria in Db mice was significantly advanced while the albumin excretion rate in non‐Db mice did not have a significant change compared to Week 16 (Figure 2b). However, rHDL treatment for 4 weeks (from Week 16 to 20) significantly blunted progression of albuminuria in Db mice even though their albumin excretion rates before the treatment was comparable (Table 2 and Figure 2b, Db vs. Db+rHDL).

**TABLE 2:** Baseline renal function of mice in non‐Db, Db, and Db+rHDL groups.

Group	ACR	GFR (μL/min/100 g BW)
Non‐Db (4M + 2F)	0.4 + 0.5	1267.3 + 224.3
Db (4M + 4F)	243.0 + 96.6***	1288.4 + 466.8
Db+rHDL (3M + 3F)	384.1 + 261.2***	974.2 + 180.1

*Note*: Data are expressed as mean ± SD. The number inside parenthesis indicates the number of mice in each group.

Abbreviations: F, female; M, male.

*** Denotes *p* < 0.001, compared to non‐Db.

Transcutaneous measurement of GFR was conducted in conscious C57BLKS/J and eNOS^−/−^ *dbdb* mice at Week 16 and 20. GFR was calculated from the sinistrin clearance rate as described in our previous publications (Chaudhari et al., 2022; Tao, Young‐Stubbs, et al., 2023). There was no significant difference in baseline GFR (at Week 16) in all 3 groups (Table 2). In non‐Db mice, the GFR was stable and there was no significant change at Week 20 compared to Week 16 (Figure 2c). Not surprisingly, GFR of Db mice was significantly decreased at Week 20 from that at Week 16 (Figure 2c), suggesting occurrence of renal insufficiency at this age. However, this GFR decline was not observed in Db mice with rHDL treatment (Figure 2c).

Taken together, these results suggest that rHDL treatment improved renal function in DKD mice.

### 3.3 Amelioration of histological impairment in Db mice with rHDL treatment

**FIGURE 3:**
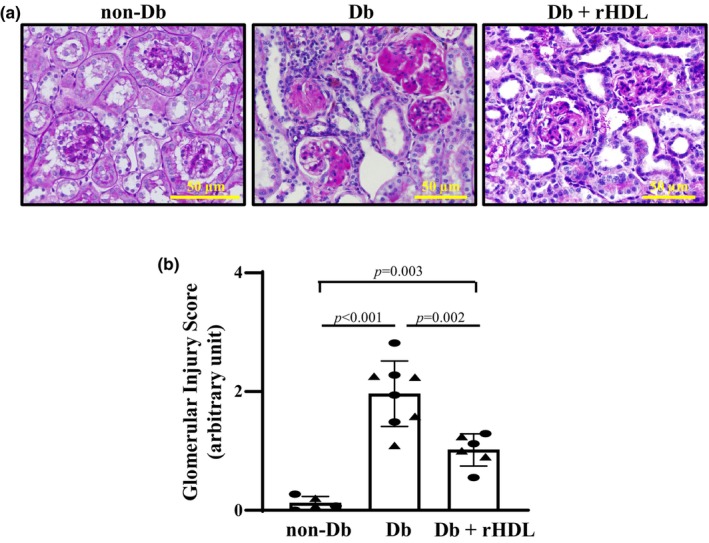
Amelioration of glomerular histopathology by rHDL treatment in mice with DKD. (a) Representative images of PAS staining from a male WT C57BLKS/J (non‐Db), eNOS^−/−^ *dbdb* (Db), and eNOS^−/−^ *db/db* + rHDL treatment for 4 weeks (Db+rHDL) mouse at Week 20. (b) Summarized glomerular injury scores from 5 (3 males and 2 females) non‐Db, 8 (4 males and 4 females) Db, and 6 (3 males and 3 females) Db+rHDL mice. About 20 glomeruli from one kidney section were counted and 5 sections were taken from one mouse. Statistical analysis: One Way ANOVA followed by Student–Newman–Keuls post‐hoc test, comparison between the groups as indicated. Solid circle represents males and solid triangle represents females.

In agreement with published studies from our group and others (Li et al., 2018; Ma et al., 2019; Zhang et al., 2012; Zhao et al., 2006), profound renal injuries were observed in eNOS^−/−^ *db/db* mice. These included substantial glomerular mesangial expansion and glomerulosclerosis, and glomerular and interstitial fibrosis. The glomerular injuries in diabetic mice were evaluated using glomerular score. As shown in Figure 3, the glomerular score of Db mice was significantly greater than that of non‐Db controls at Week 20. rHDL treatment for 4 weeks significantly reduced glomerular score in Db mice (Figure 3).

**FIGURE 4:**
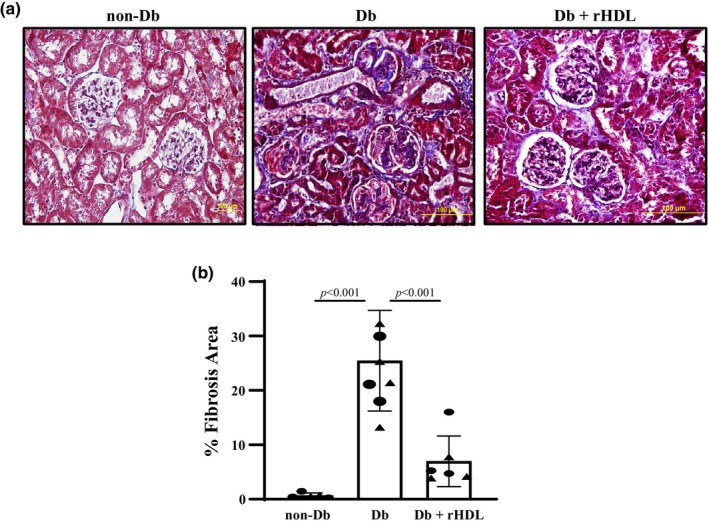
Amelioration of renal fibrosis by rHDL treatment in mice with DKD. (a) Representative Masson trichrome staining of kidney sections from a male WT C57BLKS/J (non‐Db), eNOS^−/−^ *dbdb* (Db), and eNOS^−/−^ *db/db* + rHDL treatment for 4 weeks (Db+rHDL) mouse at Week 20. (b) Semiquantitative scores of renal fibrosis from 5 (3 males and 2 females) non‐Db, 8 (4 males and 4 females) Db, and 6 (3 males and 3 females) Db+rHDL mice. About 20 glomeruli from one kidney section were counted and five sections were taken from one mouse. Fibrosis area was expressed as the percentage of the area of Masson trichrome staining in the entire area of the image. Statistical analysis: One way ANOVA followed by Student–Newman–Keuls post‐hoc test, comparison between the groups as indicated. Solid circle represents males and solid triangle represents females.

Renal fibrosis, including glomerular and interstitial fibrosis is one characteristic of DKD. Histological evidence of renal fibrosis was evaluated by staining collagen with aniline blue using the Masson trichrome staining protocol. The significant increases in aniline blue‐positive areas in kidney sections from eNOS^−/−^ *db/db* mice versus WT mice indicated marked renal fibrosis in Db mice (Figure 4). Consistent with the results presented in Figure 3, rHDL treatment for 4 weeks significantly reduced the renal fibrotic area (Figure 4).

Taken together, the results presented in Figures 3 and 4 suggest that rHDL treatment improved renal histology in mice with DKD.

### 3.4 Amelioration of podocyte injury in Db mice with rHDL treatment

**FIGURE 5:**
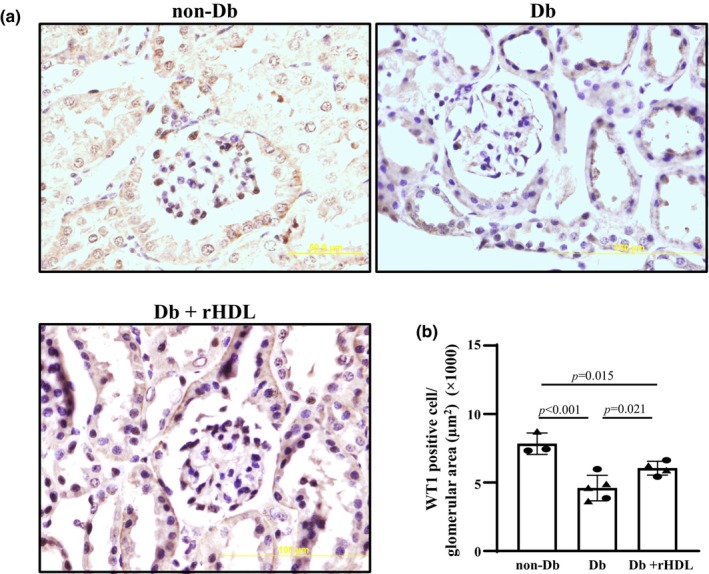
Amelioration of podocyte injury by rHDL treatment in mice with DKD. (a) Representative light microscopic images of immunohistochemistry of WT1 in kidney sections from one male non‐Db, Db, and Db+rHDL mouse at age of 20 weeks. The original magnification was ×400. (b) Quantification of WT1 positive areas calculated by [(WT1 positive cell number/glomerular area) × 100%]. Results were generated from 50 to 75 randomly selected glomeruli in five different sections per mouse in each group. Non‐Db group included 3 mice (2 males and 1 female). Db group included 5 mice (2 males and 3 females). Db+rHDL group included 4 mice (2 males and 2 females). One way ANOVA followed by Student–Newman–Keuls post‐hoc test was used for statistical analysis. Solid circle represents males and solid triangle represents females.

Podocyte death and detachment from glomerular basement membrane is one of the main manifestations of podocyte injury in DKD (Greka & Mundel, 2012; Spurney & Coffman, 2008; Tao et al., 2022; Tao, Yazdizadeh Shotorbani, et al., 2023). We quantified podocyte numbers by normalizing the number of WT1 staining cells to the glomerular area. As shown in Figure 5, WT1 positive cells in glomerulus were significantly reduced in Db mice compared to non‐Db mice. rHDL treatment for 4 weeks significantly blunted the podocyte loss in Db mice. These results are consistent with the results presented in Figures 2, 3, 4 and suggest that rHDL treatment can protect podocytes in DKD.

### 3.5 Amelioration of extracellular matrix protein accumulation by rHDL treatment in mice with DKD

**FIGURE 6:**
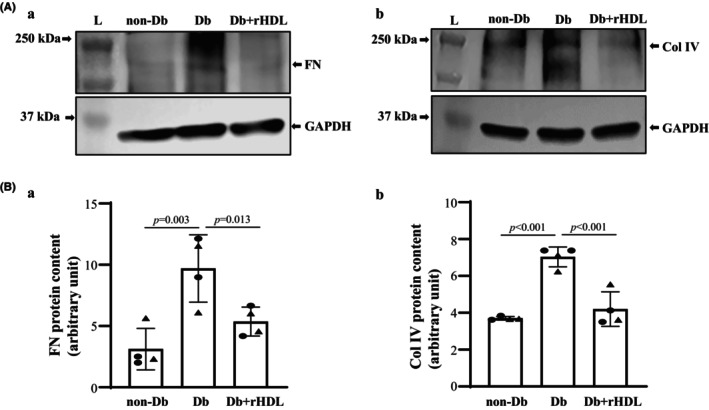
Abundance of renal cortical FN and Col IV proteins in non‐Db mice and Db mice with and without rHDL treatment. (A) Representative Western blots of renal cortical extracts, showing the protein abundance of FN (Aa) and Col IV (Ab) in the cortex of kidney from a male WT (non‐Db), eNOS^−/−^ *db/db* (Db), and eNOS^−/−^ *db/db* with rHDL treatment for 4 weeks (Db+rHDL) mouse at Week 20. GAPDH was used as the loading control. (Ba, Bb) Summary densitometric data from experiments presented in (Aa, Ab), respectively. Statistics: All groups included 4 mice (2 males and 2 females). One Way ANOVA followed by Student–Newman–Keuls post‐hoc test was used for statistical analysis, comparison between the groups as indicated. Solid circle represents males and solid triangle represents females.

Over production of extracellular matrix (ECM) proteins and deposition of these proteins in the mesangium is an important contributor to mesangial expansion in DKD (Gooch et al., 2003; Gorin et al., 2005; Schlondorff & Banas, 2009). FN and Col IV are major components of increased glomerular ECM in DKD (Cohen et al., 2001; Gooch et al., 2003; Gorin et al., 2005; Matsubara et al., 2015). We conducted Western blot using the renal cortical proteins extracted from non‐Db, Db, and Db+rHDL mice at Week 20. As shown in Figure 6, both FN and Col IV protein contents were significantly increased in diabetic mice compared to non‐diabetic controls. Treatment with rHDL for 4 weeks significantly attenuated abundance of the ECM proteins.

**FIGURE 7:**
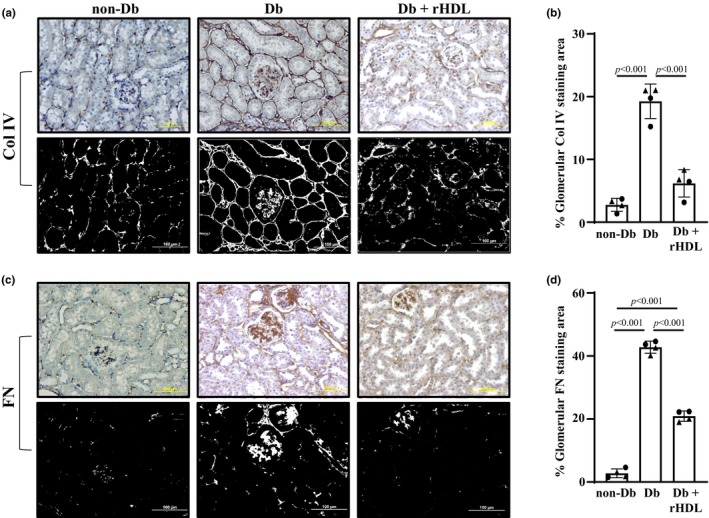
Glomerular expression of Col IV and FN in non‐Db mice, Db mice with and without rHDL treatment. (a, c) Representative light microscopic images of immunohistochemistry of Col IV (a) and FN (c). The original magnification was ×200. The upper panels in both A and B show DAB staining images and the bottom panels are black and white images converted from the same DAB images using ImageJ color deconvolution plugin function for identification of Col IV and FN (white region). (b, d) Semiquantitation of glomerular stain of Col IV (b) and FN (d) from 4 mice (2 males and 2 female) in each group. Each data point represents one mouse. For each mouse, 5 longitudinal sections of the left kidney were randomly selected and about 15–20 glomeruli from one section were randomly taken and counted. Statistical analysis: One way ANOVA followed by Student–Newman–Keuls post‐hoc test, comparison between the groups as indicated. Solid circle represents males and solid triangle represents females.

Immunohistochemistry was then used to examine expression levels of glomerular FN and Col IV proteins in the 3 group of mice at Week 20. The glomerular staining areas and intensities for both FN and Col IV were markedly increased in eNOS^−/−^ *db/db* (Db) mice compared to WT (non‐Db) mice, suggesting accumulation of ECM proteins in the glomeruli. rHDL treatment (Db+rHDL) noticeably reduced the ECM protein accumulation in Db mice (Figure 7a,b). Semiquantitation of the positively stained areas in the glomeruli showed significant increases in the expression levels of both FN and Col IV in Db mice. rHDL treatment significantly decreased the levels of both ECM proteins in Db mice even though FN level was still significantly greater than that in non‐Db mice (Figure 7b,d).

Taken together, the results from the immunohistochemistry and Western blots suggest that the rHDLs significantly suppressed production and deposition of ECM proteins in glomerular mesangium which is one of major pathologic changes contributing to the progression of DKD.

### 3.6 rHDL treatment reduced oxidative stress in mice with DKD

**FIGURE 8:**
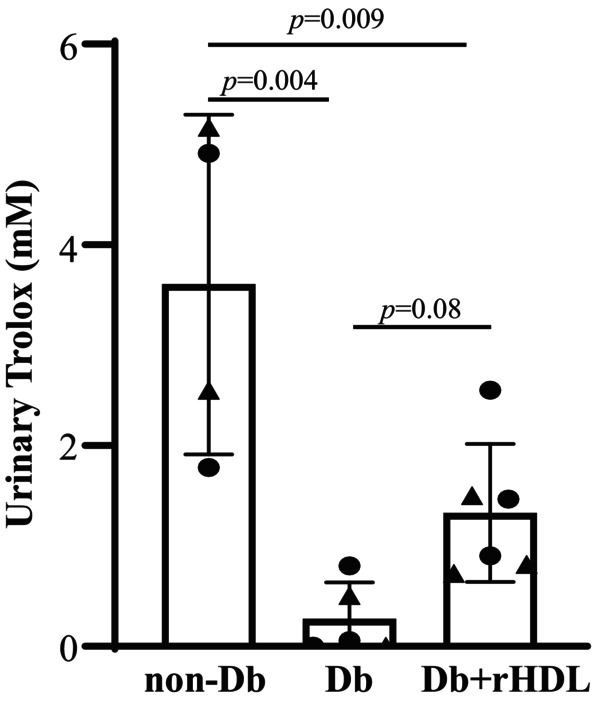
Urinary Trolox concentrations in non‐Db mice and Db mice with and without rHDL treatment. Urine samples were collected from 4 non‐Db (2 males and 2 females), 5 Db (3 males and 2 females), and 6 Db+rHDL (3 males and 3 females) at age of 20 weeks. One Way ANOVA followed by Student–Newman–Keuls post‐hoc test was used for statistical analysis, comparison between the groups as indicated. Solid circle represents males and solid triangle represents females.

Antioxidative property has been reported to be one of mechanisms for the beneficial effects of HDL (Gao et al., 2022; Kronenberg, 2018; Murphy et al., 2012; Wolkowicz et al., 2021). Oxidative stress contributes to the development of DKD (Kanwar et al., 2005, 2008). To determine whether our rHDLs could reduce oxidative stress in eNOD^−/−^ *dbdb* mice, we assessed antioxidant capacity by measuring urinary trolox level (a biomarker for oxidative stress levels) in non‐Db, Db, and Db+rHDL mice at Week 20. As shown in Figure 8, diabetes significantly reduced urinary Trolox level, indicating an increase in oxidative stress in DKD mice. rHDL treatment clearly raised the urinary Trolox level in DKD mice (0.33 ± 0.19 vs. 1.16 ± 0.28, Db vs. Db+rHDLs, mean ± SD). Although this increase did not reach statistically significant level (*p* = 0.08), the tendency to improvement of oxidative stress by rHDL treatment suggest that the rHDLs decreased oxidative stress in eNOS^−/−^ *dbdb* DKD mice.

## 4 DISCUSSION

The major findings from this study include ameliorating albuminuria, halting GFR from decline, attenuating glomerular mesangial expansion/glomerulosclerosis, ECM protein accumulation and renal fibrosis, and suppressing podocyte loss by rHDL treatment in DKD mice. These results suggest that our rHDL formulation can improve renal histology and function, and thereby, slows progression of DKD. HDL participates in the hepatic clearance of excess cellular cholesterol, that is, the reverse cholesterol transport. In diabetes patients HDL level is reduced and HDL function is impaired (Wolkowicz et al., 2021). Therefore, HDL supplementation may correct the diabetic complications due to the dyslipidemia. However, isolation of large quantities of HDL and apoA‐I (the major component of HDL) from human plasma that are functionally active is not practical. There is a need to design HDL and apoA‐I mimetics responsible for clearance of large amounts of plasma cholesterol that are small in size and amenable to organic synthesis in large quantities. In this regard, artificial constructs mimicking HDL, including apoA‐I mimetics, have been investigated for their clinical applications for decades (Heinrich et al., 2019; Kalayci et al., 2022; Kootte et al., 2015; Raut et al., 2020; Wolkowic et al., 2021). The rHDL nanoparticles developed in our laboratory possess several beneficial features. Upon dissolution in water they spontaneously assemble into a relatively small diameter spherical structure, resembling the native pre‐beta HDL particles that tend to be efficient cholesterol removers from tissues (Lacko et al., 2023). Another advantage of our rHDL nanoparticles are their close structural resemblance to circulating human HDL (Lacko et al., 2002, 2023). Additional feature is the core/surface structural arrangement of our rHDL, leaving room for a drug in the interior of the nanoparticles (Lacko et al., 2002, 2023). Over past decades, we have successfully used the rHDL formulations in patients with cancers (Lacko et al., 2007; Mooberry et al., 2016; Sabnis et al., 2012). Therefore, the rHDL formulation used in the present study is a safe therapeutic mode and has a translational potential for patients with DKD.

Although it is evident that HDL has beneficial effects on organ injury including kidney injury (McDonald et al., 2003; Moreira et al., 2014), it is also reported that HDL levels are not always associated with cardiovascular and chronic kidney disease (Barter et al., 2007; HPS2‐THRIVE Collaborative Group et al., 2014; Kronenberg, 2018; Voight et al., 2012). The discrepancy is not surprising considering the complexity and heterogeneity of HDL particles, as well as their components and their involvement in cholesterol efflux and reverse cholesterol transport. In addition, HDL disturbances in the functionality, and especially the reverse cholesterol transport might be different between various stages of kidney impairment. The major component of rHDL nanoparticles used in the present study is apoA‐I, which is the major apolipoprotein of almost all plasma HDLs and possesses the beneficial effects of HDL (Gao et al., 2022; McDonald et al., 2003; Sabnis et al., 2012; Wolkowicz et al., 2021). Our results show that rHDL treatment significantly alleviated glomerular injury, ECM accumulation, and renal fibrosis, and halted progression of albuminuria and GFR decline in mice with DKD. Therefore, our rHDL formulation can improve renal histology and function, and thereby slows progression of DKD.

In addition to beneficial effects on many diseases, HDL nanoparticles can also be used as carriers to deliver a specific drug to target organs (Gao et al., 2022; Lacko et al., 2023). We have previously demonstrated that our rHDL nanoparticles could successfully deliver anti‐cancer chemicals to tumors in mice and humans (Lacko et al., 2023; Sabnis et al., 2017). The two properties (tissue/organ protection and nanocarriers) of rHDLs double their advantages when they are used to treat a disease. We reason that the rHDLs used in the present study can be used as nanocarriers to deliver an anti‐diabetic medicine to patients with DKD. Further study is warranted to explore this possibility.

It is noted that our rHDL treatment prevented progressive decline of GFR, but did not significantly improve BUN level in DKD mice (Figure 1a,c). Although BUN is freely filtered and essentially reflects GFR, their relation to GFR is not a straight line, but rather a parabolic curve. For instance, BUN level still remains normal when there is a 50% GFR lost in human (Hosten, 1990). Also, the level of BUN is affected by dietary and physiologic conditions not related to renal function, such as protein intake, endogenous protein catabolism, state of hydration, and hepatic urea synthesis (Hosten, 1990). In addition, the BUN level is also associated with renal tubular function because urea can be secreted and reabsorbed by renal tubules (Sands, 2007). It is known that the urea absorption in the collecting duct is regulated by several hormones and urinary flow rate (Schrier, 2008). Hence, there are several limitations on the usefulness of the BUN when assessing renal/glomerular function and the limitations could explain the discrepant responses on rHDL treatment in DKD mice between BUN and GFR.

HDL has been well known to have potent anti‐inflammatory and antioxidative properties (Gao et al., 2022; Kronenberg, 2018; Murphy et al., 2012; Wolkowicz et al., 2021). Both inflammation and oxidative stress contribute to renal injury of DKD (Gorin, 2013; Graham et al., 2011; Ha et al., 2008; Verhave et al., 2013; You et al., 2013). Although it was not thoroughly examined in the present study, the renoprotective mechanisms of our rHDLs in DKD might involve a decrease in oxidative stress because the urinary Trolox level in rHDL‐treated eNOS^−/−^ *dbdb* mice had a clear tendency to decrease compared to eNOS^−/−^ *dbdb* mice without treatment (Figure 8). Nevertheless, it would be interesting to comprehensively examine whether and how rHDLs suppress one or more inflammatory pathways and/or redox imbalance. To this end, further studies need to be carried out to assess the activity/abundance of inflammatory cytokines/chemokines and of enzymes related to reactive oxygen species production/degradation/scavenging in kidney in response to rHDL treatment.

There are a few of limitations in the present study. First, the rHDLs were administered into the eNOS^−/−^ *dbdb* mice at Week 16 when overt DKD had developed (Ma et al., 2019; Zhao et al., 2006). Therefore, the results from the present study suggest a therapeutic, but not a preventative potential. To answer the latter question, we may apply the rHDLs into eNOS^−/−^ *dbdb* mice at early age, such as at Week 4 when DKD symptoms has not manifested (Ma et al., 2019; Zhao et al., 2006). Second, the lipid panels were not analyzed in this study. It is known that in patients with insulin resistance and type 2 diabetes, plasma lipid and lipoprotein abnormalities are common (Luscher et al., 2003). Whether our rHDL treatment could correct or improve the diabetic dyslipidemia in the DKD mice is not known and is worthy to study further. Lastly, all control mice (WT C57BLKS/J) in this study were male, but Db mice (eNOS^−/−^ *dbdb*) included both males and females. Although our previous studies (Ma et al., 2019; Tao, Young‐Stubbs, et al., 2023) demonstrated no sex differences in renal function and pathology in both WT C57BLKS/J and eNOS^−/−^ *dbdb* mice, possible differences in susceptibility to rHDL treatment between males and females can not be ruled out.

In summary, the present study showed a beneficial role of our rHDL formulation in treating mice with DKD by ameliorating renal histological and functional injury. Therefore, our rHDL treatment could slow progression of DKD. The renoprotective effects of our rHDLs and their potential to be anti‐diabetic drug carriers provide an alternative therapeutic option for patients with DKD. Since the effectiveness of current treatments for DKD, including use of antihypertensive medications and renin‐angiotensin system inhibitors, and glycemic control is only modest (Pavkov et al., 2009), our findings are in compliance with currently urgent needs for a new and effective treatment for patients with DKD.

## AUTHOR CONTRIBUTIONS

Ma R conceived and designed the study, analyzed data; prepared most figures, and drafted the manuscript. Tao Y performed most experiments, interpreted the results of experiments, and prepared some figures; Lacko AG and Sabnis NA prepared rHDL formulations. Paromita D analyzed some histology data and Crowe N conducted some histology experiments. Ibrahim D bred and genotyped eNOS^−/−^ *dbdb* mice. Zhou Z was consulted with statistical analysis. All authors approved the final version of this manuscript.

## FUNDING INFORMATION

The work was supported by National Institutes of Health Grant R01s (NIH/NIDDK, DK115424‐01 to R. Ma), the Translational Project Award from American Heart Association (20TPA35500045, to R. Ma), The Seed grant from Department of Physiology and Anatomy, University of North Texas Health Science Center (to R. Ma and A. Lacko), and American Heart Association Predoctoral Fellowship (22PRE903925, to Y. Tao).

## CONFLICT OF INTEREST STATEMENT

The author declares no conflicts of interest.

## ETHICS STATEMENT

All animal procedures were conducted under protocols approved by the University of North Texas Health Science Center (UNTHSC) Institutional Animal Care and Use Committee. All experimental protocols involving animal usage and animal care were in accordance with the American Association for Assessment and Accreditation of Laboratory Animal Care (AAALAC) and National Institutes of Health (NIH) guidelines.
